# Total Energy Expenditure and Physical Activity Profiles Among Reindeer Herders and Office Workers of Northern Finland

**DOI:** 10.1002/ajhb.70188

**Published:** 2025-12-28

**Authors:** Ville Stenbäck, Minna Turunen, Päivi Soppela, Karl‐Heinz Herzig, Cara Ocobock

**Affiliations:** ^1^ Department of Anthropology University of Notre Dame South Bend Indiana USA; ^2^ Research Unit of Internal and Biomedicine, Faculty of Medicine University of Oulu Oulu Finland; ^3^ Arctic Centre University of Lapland Rovaniemi Finland; ^4^ Department of Pediatric Gastroenterology and Metabolic Diseases, Pediatric Institute Poznan University of Medical Sciences Poznan Poland; ^5^ Medical Research Center (MRC) Oulu University Hospital Oulu Finland; ^6^ Biocenter Oulu University of Oulu Oulu Finland

**Keywords:** cold climate, FLEX‐HR, physical activity, RMR, total energy expenditure

## Abstract

**Objectives:**

In the Arctic, climate change increases extreme weather events and unpredictability, affects food chains, increases transportational needs, and decreases physical activity (PA) and estimated total energy expenditure (eTEE). Thus, understanding how climate change affects inhabitants of different environments is increasingly important. The reindeer herders of Finnish Lapland are exposed to changing weather conditions year‐round and have a highly physically demanding occupation.

**Materials and Methods:**

We studied eTEE, physical activity level (PAL), and PA profiles of reindeer herders (*n* = 10) and office workers (*n* = 13) in the Inari Municipality area in Finland February of 2023 as a pilot study. eTEE was estimated using the FLEX‐HR method and PA parameters were measured using accelerometry.

**Results:**

We did not observe any statistically significant differences between the occupations, but there was a trend of reindeer herders being heavier, having more muscle mass, and greater eTEEs relative to office workers. eTEE for herders was 2887.1 ± 1675.4 and 2038.9 ± 593.1 kcal/day for office workers.

**Discussion:**

With the seasonal nature of reindeer herding, February is a period of relative ease following the physically demanding round‐up period that lasts from September to January, which may explain these results. Furthermore, large variation in the main variables of eTEE and PAL highlights the need for a larger study population. Therefore, a seasonal assessment of PA and eTEE patterns in this unique population where herders and office workers live close to one another and share multiple lifestyle aspects in the rapidly warming Arctic is needed.

## 1 Introduction

The dual burdens of increasing rates of obesity and environmental unpredictability negatively impact human health and well‐being. As such, studying how climate change impacts health across a wide variety of environments and developing environment‐specific prevention strategies is essential. Worldwide, the age‐standardized prevalence of obesity has increased from 4.6% in 1980 to 14.0% in 2019, tripling since 1975 (Boutari and Mantzoros 2022). There are approximately 650 million people who have obesity and 2 billion who are considered overweight. In the Americas and Europe, the situation is worse where 22.4% and 20% of the population, respectively, have obesity (Boutari and Mantzoros 2022). There is a wide range of population variation, but the rates of obesity are rising steadily across the globe, with an accelerated increase during the last few decades (Boutari and Mantzoros 2022; Gildner and Levy 2021). Overweight and obesity are known risk factors for multiple noncommunicable diseases such as cardiometabolic or neurodegenerative diseases and several cancers. The main strategies for the prevention of obesity are education, a healthy diet, and sufficient physical activity (PA) (Fruh 2017).

Together with growing waistlines due to numerous social, political, and economic factors, the climate is changing. Direct consequences of climate change include rising temperatures and sea levels, increased extreme weather events, and unpredictable changes in precipitation (Bolan et al. 2024). The Arctic is warming almost four times faster than the global average and some specific areas in the Arctic Ocean up to seven times more. The pace of warming has been 0.73°C per decade in the Arctic versus the global average of 0.19°C (Rantanen et al. 2022). The indirect effect of global warming with obesity is bidirectional: with rising temperatures, people experience less adaptive thermogenesis and decrease their PA, reducing total energy expenditure (TEE, kcal/day), relying more on mechanized transportation and creating more emissions, further fueling climate change (Koch et al. 2021). Alterations in PA due to climate change are not the only factors that could lead to increased rates of obesity. Climate change also affects the availability of locally grown foods, increases the caloric value and level of processing in diets, and increases transportation costs (McGrosky et al. 2025). Together with urban expansion and factory farming, the cycle feeds itself further through rising temperatures and extreme weather events (Gildner and Levy 2021). In order to study this intertwined phenomenon, more anthropological research is needed to consider the diverse socio‐ecological patterns of TEE and PA among populations more acutely affected by climate change across a variety of environments.

TEE is the amount of energy that the human body spends during a 24‐h period, and it consists of resting metabolic rate (RMR, kcal/day, ~60%–80% of TEE), thermic effect of food (~10% of caloric intake), thermoregulation, and PA or activity energy expenditure (~15%–30% of TEE) (Heydenreich et al. 2017). Variations in TEE are due to variations in RMR and differences in PA, which is regarded as the most modifiable portion of TEE (Neilson et al. 2008). RMR is the energy required for basic metabolic processes such as maintaining body temperature and the function of vital organs such as the liver, brain, and heart (Ndahimana and Kim 2017; Young and Castellani 2001). PA varies the most at intra‐ and interpersonal levels. In sedentary people, PA can be roughly half that of RMR, but in highly active people, it can be over 2 times the rate of RMR (Ndahimana and Kim 2017). Intensity, duration, and frequency of an activity all affect PA. In cold environments, PA has been shown to decrease thermoregulatory costs despite increasing blood flow to the skin and thus increasing heat dissipation (Young and Castellani 2001; Ocobock 2016).

Physical activity levels (PALs) are reported as TEE/RMR and represent the proportion of PA in TEE. PAL range from a minimum of 1.1–1.2 to a maximum of 2.0–2.5 in a sustainable lifestyle (Westerterp 2013). PAL values of ≥ 1.0 and < 1.4 are considered sedentary, ≥ 1.4 and < 1.6 low active, ≥ 1.6 and < 1.9 active, and ≥ 1.9 and < 2.5 highly active (Trumbo et al. 2002). PA can be further assessed by examining time spent sedentary, in light intensity PA (LPA), in moderate‐intensity PA (MPA), and in vigorous intensity PA (VPA). Thus, PA behavior can be studied without direct measurement of TEE or RMR.

Advances in wearable technology have made it possible to estimate TEE and PA with the use of accelerometry, pedometry, and heart rate monitoring or a combination of these among free‐living populations. Commonly, accelerometry and HR monitoring are utilized simultaneously in the FLEX‐HR method (Hiilloskorpi et al. 2003). These methods do not yield as accurate TEE data as doubly labeled water (DLW) or indirect calorimetry (IC), but the relatively low cost and low participant burden make them ideal for population‐scale measurements (Ndahimana and Kim 2017). The accuracy and validity of the accelerometry and HR measurements can be increased with calibration or by using them alongside DLW or IC.

TEE and PAL have been studied around the globe, with an emphasis on developed countries. Dugas and colleagues performed a meta‐analysis comparing TEE and PAL of adults in industrialized countries vs. developing countries categorized using the human development index (HDI). The mean TEE for men was 3035 ± 48 kcal/day and 2366 ± 24 kcal/day for women. Mean PAL for men was 1.80, and 1.71 for women (Dugas et al. 2011). Surprisingly, no association of HDI status with either TEE or PAL was reported, suggesting that in this study, cultural and financial differences are not determinative for TEE and PAL (Dugas et al. 2011). Worldwide PA parameters apart from PAL, such as step count and intensity‐based classifications, have been studied extensively (Stenbäck et al. 2024, 2021). Tudor‐Locke and colleagues examined PA metrics in the USA (NHANES, 2005–2006). In total, participants averaged 6564 daily steps, spent 56.8% of the waking day in sedentary time, 23.7% in low intensity, 16.7% in LPA, 2.6% in MPA, and 0.2% in VPA. Overweight participants and those with obesity were less active and had less VPA than normal weight people (Tudor‐Locke et al. 2010). Similar numbers have been reported from Nordic studies, with slightly more MVPA and higher daily step counts (Kuster et al. 2021; Dohrn et al. 2024).

Despite these large studies assessing time‐wise PA variables, there is a lack of TEE and PAL measurements among cold climate populations that follow more traditional lifestyles. However, there is a growing body of work examining TEE among warm climate traditional populations (Christopher et al. 2019; Pontzer et al. 2012). These studies suggest that human TEE may have evolved as a physiological trait rather than a cultural one, which explains it being relatively stable across populations. Snodgrass et al. (2006) conducted one of the few studies in a cold climate population investigating TEE in Yakut people from the Sakha Republic of Russia. They found that men had higher activity levels than women after body size adjustment likely due to differences in work/household activities (TEE: 3103.01 ± 194.07, 2299.2 ± 124.7 kcal/day; PAL: 1.68 ± 0.33, 1.50 ± 0.20) (Snodgrass et al. 2006). PAL was relatively low overall when compared to other populations. Sellers et al. (2022) studied nomadic or semi‐nomadic herders from Tuva, a Republic in southern Siberia within the Russian Federation (Tuvan pastoralists), and reported high TEE values of 3224 ± 318 kcal/day with a PA time of 609 ± 90 min/day, while PAL was 1.89 ± 0.34. Furthermore, daily PA consisted of 475 ± 91 min/day in LPA, 125 ± 60 min/day in MPA, and 8 ± 11 min/day in VPA (Sellers et al. 2022). In a similar climate, our earlier work with Finnish reindeer herders in autumn 2018 revealed high TEE values of 4183 ± 709 kcal/day. There were no significant differences between females and males once differences in body size were considered (Ocobock et al. 2020). These three studies represent the majority of work on TEE among cold climate populations to date.

The reindeer herders of Finnish Lapland follow a traditional, although modernized, lifestyle in the most rapidly warming area in the world. The aim of this study was to describe and compare TEE, PAL, and PA profiles between reindeer herders and office workers from the Inari municipality, Finland (68.9° N latitude) during the coldest time of the year. We hypothesized that herders have higher PAL, MVPA, and TEE due to the physical demands of their occupation. This study adds to a growing body of literature that evaluates human energy expenditure and PALs across a wide variety of environments. This work will help to elucidate the range of TEE and PAL variations among populations across the globe, and help us to better understand how TEE, PAL, and the environment interact—a critical area of research in our rapidly changing climate.

## 2 Participants and Methods

A total of 36 participants enrolled for the study and 23 participants had valid PA and FLEX‐HR data. 13 reported their occupation as office workers and 10 as reindeer herders (Table 1). Participants for this study were recruited using multiple media platforms in the Inari municipality, Finland (68°54′18″ N, 027°01′49″ E). The study was approved by the University of Notre Dame Internal Review Board (Protocol# 21‐09‐6826), the regional medical research ethics committee of the Wellbeing services county of North Ostrobothnia, Oulu, Finland (POHDE EETTMK: 49/2022), and the Sámi Parliament (LAUSUNTO 25.5.2021, Dnro:34/D.a/2021). All participants were provided with a detailed information sheet about the study, and they gave their written consent in Finnish (Heikkilä et al. 2024). A background questionnaire was completed to exclude people with conditions or medication which could directly influence EE. We excluded individuals from participating if they were older than 65 or under 18, they were pregnant or lactating, had severe hypertension, used beta‐blockers, had a body mass index (BMI) < 19, had cardiopulmonary issues, had Raynaud's disease, and/or had metabolic issues such as diabetes.

**TABLE 1:** The ranges, means and standard deviations of anthropometric, energy expenditure (EE), and physical activity (PA) variables.

	All (*N* = 23)		Office (*n* = 13)		Herder (*n* = 10)		Sig
	Min.–Max.	Mean ± SD	Min.–Max.	Mean ± SD	Min.–Max.	Mean ± SD	
*Anthropometry*							
Age	24–64	45.5 ± 12.2	36–64	48.2 ± 9.8	24–64	41.9 ± 14.6	0.313
Height (cm)	140.7–188.4	164.5 ± 10.9	154.8–179.4	164.1 ± 6.3	140.7–188.4	165.1 ± 15.5	0.976
Weight (kg)	47.2–110.6	69.7 ± 16.8	51.4–82.3	65.1 ± 7.6	47.2–110.6	75.6 ± 23.3	0.693
BMI	20.6–33.0	25.4 ± 3.3	20.7–26.7	24.1 ± 1.7	20.6–33.0	27.1 ± 4.2	0.148
Fat%	26.7–45.6	34.6 ± 5.2	26.7–41.8	34.2 ± 4.7	28.2–45.6	35.1 ± 6	0.738
Waist (cm)	70.6–115.3	85.2 ± 12.7	70.6–92.5	80.5 ± 7	74–115.3	91.2 ± 16.1	0.115
Hip (cm)	83.4–108.2	93.4 ± 7.1	83.4–108.2	91.8 ± 7	84.3–105.2	95.5 ± 7	0.232
Waist‐to‐hip ratio	0.76–1.10	0.91 ± 0.10	0.76–1.05	0.88 ± 0.08	0.80–1.10	0.95 ± 0.11	0.137
FFM	29.2–77.1	45.6 ± 12.1	34.6–53.1	42.7 ± 5	29.2–77.1	49.4 ± 17.1	0.563
SMM	12.6–38	21.1 ± 6.6	15.9–25.6	19.5 ± 2.8	12.6–38	23.3 ± 9.3	0.693
*EE and PA*							
eTEE (kcal/day)	1536.3–7172.0	2407.7 ± 1234.9	1536.3–3445.2	2038.9 ± 593.1	1557–7172.0	2887.1 ± 1675.4	0.208
Steps/day	6011.4–20338.6	11 456 ± 3300	7165.7–15841.0	11 184 ± 2757.9	6011.4–20338.6	11809.6 ± 4029	0.976
Sedentary (min/day)	312.1–1174.9	793.4 ± 239.8	409.7–1174.9	847.5 ± 198.8	312.1–1061.1	723.1 ± 279.7	0.313
LPA (min/day)	69.7–288.9	163 ± 52.2	101–222.1	153.7 ± 38	69.7–288.9	175.2 ± 66.7	0.376
MVPA (min/day)	46.1–190	99.3 ± 37.7	46.1–190	111.2 ± 42.5	53.2–116.4	83.8 ± 24.3	0.115
Sedentary %	49–88.9	74 ± 8.6	55.3–88.9	75.3 ± 8.2	49–80.4	72.3 ± 9.3	0.522
LPA %	7.6–36.4	16.1 ± 6.4	7.6–30.0	14.4 ± 5.5	12.1–36.6	18.4 ± 7.1	0.049
MVPA %	3.5–16.2	9.9 ± 3.9	3.5–16.2	10.3 ± 4.2	4.8–14.6	9.2 ± 3.4	0.446
RMR (kcal/day)	1011.0–2554.0	1675.9 ± 466.6	1101.0–2554.0	1528.6 ± 457.7	1338.0–2505.0	1867.3 ± 425.1	0.101
PAL	1.03–2.86	1.42 ± 0.41	1.05–1.99	1.37 ± 0.31	1.03–2.86	1.47 ± 0.52	0.738

*Note:* Mann–Whitney *U* tests were used to study differences in each variable between occupations.

Abbreviations: BMI = body mass index, eTEE = estimated total energy expenditure, FFM = fat‐free mass, LPA = light physical activity, MVPA = moderate to vigorous physical activity, PAL = physical activity level, RMR = resting metabolic rate, SMM = skeletal muscle mass.

The majority of our participants were female; three of the herders and two of the office workers were male. Participants self‐reported their sex. All participants in this study came from the Inari municipality in Northern Finland, subjected to the same weather conditions though different exposure levels due to their occupational demands. Office workers were employed in an indoor setting mainly in tourism, services, and administration. Reindeer herding consists of hard physical work outdoors regardless of the weather conditions and involves collecting and moving reindeer, working in round‐ups, slaughtering the animals, processing meat, daily feeding, and monitoring the safety of the animals (Turunen et al. 2021). Reindeer herding tasks vary seasonally and include both traditional and modern practices and technology.

### 2.1 Anthropometry and RMR

Height (cm), body mass (kg), and hip and waist circumferences (cm) were recorded within one decimal accuracy. Body composition variables (skeletal muscle mass [SMM], fat free mass [FFM], % body fat) were determined using bioelectrical impedance (RJL systems, Clinton, MI, USA). Participants were asked to refrain from alcohol the day before the measurement. Participants were placed in a supine position on a bed, and electrodes were arranged on the dorsum of the wrist, middle finger, ankle, and middle toe on the right and left sides of the body. Reactance and resistance were recorded, and variables calculated with standard BIA‐103 equations. BMI was calculated with the following equation: body mass (kg)/height (m)^2^. RMR was evaluated using IC in the morning when participants were overnight fasted with the Cosmed K5 mobile unit (Cosmed, Chicago, IL, USA). The detailed procedure has been described in our previously (Ocobock et al. 2020).

### 2.2 PA Measurement

Participants wore an ActiGraph GT3X+ (ActiGraph LLC, Pensacola, FL, USA) on their nondominant wrists and a chest strap heart rate monitor (Polar Electro Oy, Kempele, Finland) for 10 days. Data was extracted and analyzed using Actilife v6.13.6 software. The analysis was conducted in 60 s epochs. Wear time and non‐wear time were detected using the method developed by Troiano et al. (2008). A valid measurement period was determined to be 5 days or more. LPA, MPA, and VPA were generated utilizing the cut‐offs developed for nondominant wrist wear by Montoye et al. (2020), since the ActiLife's preset equations have been developed with hip‐worn devices and/or for children. MVPA was calculated as a sum of MPA and VPA. Estimated TEE (eTEE) was calculated from HR data using the FLEX‐HR method and TEE estimation equations published by Hiilloskorpi and colleagues, which were significantly correlated with DLW‐measured TEE in our earlier work with similar population (Hiilloskorpi et al. 2003; Ocobock et al. 2022). eTEE was calculated as kcal/min and all values less than participants' RMR were replaced with the IC determined RMR as kcal/min to eliminate the effect of HR values less than 40. After data cleaning, values were transformed into kcal/day according to the valid individual measurement length. PAL was calculated as eTEE/RMR.

### 2.3 Statistics

All statistical analyses were conducted and figures generated using the IBM SPSS Statistics 29.0.2.0 software. Normality was tested using the Kolmogorov–Smirnov test. The nonparametric Mann–Whitney test was utilized to study the differences in absolute eTEE, anthropological variables and PA behavior between sexes and occupations. Multiple linear regression modeling was utilized to study the relationship of body mass, FFM, sex and occupation with eTEE. Both models included eTEE and occupation, and either FFM or body mass was further added. We did not separately include sex, since FFM and body mass control for body size, and the number of males was low. *p* value of 0.05 was considered as a level of statistical significance. Results are presented as mean ± SD.

## 3 Results

### 3.1 Anthropometry

No statistically significant differences were observed between the occupations in anthropometric measures (*p* > 0.05), but there was a trend of herders being heavier (65.1 ± 7.6 vs. 75.6 ± 23.3), having more SMM (19.5 ± 2.8 vs. 23.3 ± 9.3), and greater BMI (24.1 ± 1.7 vs. 27.1 ± 4.2) (Table 1).

### 3.2 eTEE and PA

We observed no difference in eTEE between office workers and reindeer herders (*p* = 0.208). We performed linear regression analyses between eTEE, occupation, and FFM or body mass to see whether taking body size into consideration affected the association between eTEE and occupation. In the regression models, occupation was not a significant predictor for eTEE, but FFM and body mass were. The model that included eTEE, occupation (standardized *β* = 0.182, *p* = 0.310), and FFM (standardized *β* = 0.585, *p* = 003) had an adjusted *R* ^2^ of 0.435. The other model with eTEE, occupation (standardized *β* = 0.170, *p* = 0.360), and body mass (standardized *β* = 0.564, *p* = 0.006) had an adjusted *R* ^2^ of 0.348. When comparing PAL and more specific PA variables between the occupations, we observed no significant differences in any of the studied variables (*p* > 0.05), except on the percentage proportion of LPA (*p* = 0.049) (Table 1). However, there was a trend of herders having higher absolute eTEE (2038.9 ± 593.1 vs. 2887.1 ± 1675.4 kcal/day).

## 4 Discussion

We investigated PA behavior and eTEE among office workers and reindeer herders living in Inari municipality of Finland (68.9° N latitude). We hypothesized that herders, due to their physically demanding occupation, would have higher eTEE, a higher PAL, and more time spent in PA, especially in the MVPA category compared to office workers. We observed no significant differences between the occupations in eTEE, PAL, or in any of the more specific PA parameters, except in percentage proportion of LPA. However, while not significant, we observed a trend towards herders having higher absolute eTEE and PAL. Regression analysis confirmed that occupation was not a significant predictor for eTEE, but FFM and body mass were. Overall, the ranges of PA variables and eTEE were high and standard deviation large, which largely explains why we cannot see statistically significant differences between the occupations. However, there are several potential reasons for this high degree of variation.

The reindeer herding year starts on May 31 and the summer is spent by fixing fences, making hay for the reindeer, and marking the newly born calves by bringing the herd together for 2–3 weeks. In autumn, reindeer are gathered for the round‐up, sorted, counted, some are slaughtered, and the resulting meat processed for sale. In winter, additional feeding is conducted either in the forests, fells, or in fenced areas. During spring, new calves are born. The most labor‐intensive months for the herders are June and July when the newborn calves are marked, and especially during the round‐up period lasting from the end of September to January (Paliskuntain yhdistys 2025). These seasonal differences in herding tasks could partly explain why we did not observe significant differences between the herders and office workers in eTEE and PAL or in the more specific PA parameters as we measured eTEE during a less labor‐intensive period in the herding year.

The trend toward higher eTEE in herders can be due to their being heavier with more SMM. In addition, herders had a slightly higher PAL, possibly from reindeer feeding activities. Furthermore, the use of snowmobiles over skiing in herding has become the standard since the 1960's. This shift toward snowmobiles has decreased the physical demands of herding during the winter months; in addition to the use of ATVs, helicopters, and drones in summer and autumn (Turunen et al. 2021). Since these measurements were performed in February 2023, which was 2.4°C warmer than is typical in February (Finnish Meteorological Institute 2025), no “extreme” cold conditions were experienced. With climate change, the Arctic already has and is forecasted to experience more warm periods during winter, delayed snow cover, and more icing events. For the reindeer, this leads to difficulties in natural foraging, thus increasing the need for supplemental feeding either in the forest or gathering the reindeer into enclosures for feeding. This chain of events leads to increasing costs and work efforts for the herders (Ocobock et al. 2023). This additional workload could also lead to increased PAL and eTEE. Furthermore, thawing and icing cycles create difficulties in skiing and snowmobile use. Some herders perform the additional feeding by taking the feed to the forest while others gather the herd to enclosures. These different approaches could result in varying energetic needs as observed in this study.

Office worker behavior may also explain why there is little difference in PA variables or eTEE. The office workers could be more likely to live closer to their jobs and in towns, enabling walking, biking, or kick sledding rather than using a car. The office workers might have a broader range of hobbies due to more leisure time in their lives. Overall, within the Finnish adult population (age 18–64), males spend 41 and females 36 min on average on sport, exercise and outdoor activities weekly, not including transportation. Sports most often reported are walking (54%), bicycling (26%), gym use (22%), and cross‐country skiing (20%) (Koski et al. 2018). However, these surveys did not control for regional differences, such as between southern and northern areas, nor between occupational groups. In Finland, cross‐country skiing is an important means of transportation and is part of the cultural heritage, which is allowed to be performed in almost every forest, on frozen lakes, and other uninhabited areas. Pouta and colleagues found that people living in more rural areas, such as the municipality included in this study, have a higher probability of taking part in cross‐country skiing. However, they also reported that higher mean annual temperature had a negative effect of participation frequency, and the authors forecasted that the proportion of skiers and frequency of skiing will decrease by at least 20% by 2080 if the current climate warming trend continues (Pouta et al. 2009).

Comparing eTEE to other populations with similar livelihoods to the reindeer herders in this study, eTEE was similar to Sakha herders in Yakutia. We observed PAL levels of 1.42 ± 0.41 (office 1.37 ± 0.31, herder 1.47 ± 0.52). On average, these PAL levels are considered as low active (≥ 1.4 < 1.6) (Trumbo et al. 2002) and were similar to observed PAL among the Sakha (1.50 ± 0.20 women, 1.68 ± 0.33 men), but lower than Tuvan pastoralists (1.89 ± 0.34) of southern Siberia (Pontzer et al. 2012; Snodgrass et al. 2006; Sellers et al. 2022). This is interesting since mean yearly temperatures in Yakutia are lower than in Finnish Lapland (~−7.5 vs. ~−1) and the Sakha herders would have greater thermoregulatory pressure, but that did not affect eTEE (Finnish Meteorological Institute 2025; Climate Data 2025). However, the Sakha participants were largely factory workers and did not follow a traditional lifestyle. The reduced activity among the reindeer herders during the Finnish winter may drive the eTEE similarity between these two groups. However, the difference between the Finnish herders and the Tuvan pastoralists requires further elaboration. The differences in the HDI between Finland and Russia (0.942 vs. 0.821) (United Nations Development Programme 2025) could potentially explain some of the differences we see between the Finnish population and the Tuvan pastoralists (Snodgrass et al. 2006). The HDI consists of three dimensions: health (life expectancy at birth), education (mean of years in schooling) and standard of living (gross national income per capita). Our population has potentially adopted more modern technology and has better access to more expensive means of transportation such as snowmobiles, ATVs, helicopters, and drones. Apart from direct effects of herding activities, differences in housing, heating, nutrition, and access to medical care that most likely exist may be leading to the observed eTEE differences between these populations. Additionally, Tuvan pastoralists follow a nomadic lifestyle that Finnish and Sakha do not, which could explain the higher eTEE (Sellers et al. 2022).

## 5 Limitations

The main limitation to this study is the low sample size, especially that of males. This limits the power of our statistical calculations and could be the underlying reason we do not see significant differences. Female herders tend to participate in less physically demanding herding tasks than male herders and are more likely to work in herding part‐time (Turunen et al. 2026). Hence, comparisons with other populations are relatively difficult. The number of participants in these studies needs to be increased to better shed light on the typical levels and degree of variation in eTEE and PAL parameters. This is a cross‐sectional study in a location that has dramatic seasonal changes. Data collected among this population across seasons will help to paint a more nuanced picture of eTEE and PAL variation. We also did not ask the participants about their hobbies or activities, which might in the future help to explain their eTEE and PAL patterns. To better understand the variation in eTEE and PA parameters and to introduce covariates into the analyses with sufficient statistical power, we are currently doing longitudinal, seasonal measurements with a greater number of participants.

## 6 Conclusion

There were no significant differences in eTEE, PA profiles, or PAL between office workers and reindeer herders of the Inari municipality of Finland during February 2023. During this time of the year, the energy expenditure or PA profiles of reindeer herders do not significantly differ from the office workers. In February, herder activities do not significantly differ from those of office workers. We observed a trend towards herders having higher eTEE and PAL, while being heavier and having more SMM. Furthermore, this study highlights the need for longitudinal studies with sufficient participant numbers to capture the effect of seasonality on energetic needs of herding and living in the rapidly warming Arctic. Such longitudinal data will help elucidate the ways in which climate change is altering eTEE and PAL patterns.

## Author Contributions

**Ville Stenbäck:** conceptualization (equal), data curation (equal), formal analysis (lead), investigation (equal), methodology (equal), writing – original draft (equal), writing – review and editing (equal). **Minna Turunen:** investigation (equal), project administration (equal), resources (equal), writing – review and editing (equal). **Päivi Soppela:** investigation (equal), project administration (equal), resources (equal), writing – review and editing (equal). **Karl‐Heinz Herzig:** funding acquisition (equal), resources (equal), writing – review and editing (equal). **Cara Ocobock:** conceptualization (equal), investigation (equal), methodology (equal), project administration (equal), resources (equal), supervision (equal), writing – review and editing (equal).

## Funding

This work was supported by the University of Notre Dame, University of Oulu, and University of Lapland.

## Ethics Statement

The study was approved by the University of Notre Dame Internal Review Board (Protocol# 21‐09‐6826), the regional medical research ethics committee of the Wellbeing services county of North Ostrobothnia, Oulu, Finland (POHDE EETTMK: 49/2022), and the Sámi Parliament (LAUSUNTO 25.5.2021, Dnro:34/D.a/2021).

## Conflicts of Interest

The authors declare no conflicts of interest.

## Data Availability

The data that support the findings of this study are available on request from the corresponding author. The data are not publicly available due to privacy or ethical restrictions.
